# An Organic Microcavity Laser Amplifier Integrated on the End Facet of an Optical Fiber

**DOI:** 10.3390/nano14151314

**Published:** 2024-08-04

**Authors:** Meng Wang, Zhuangzhuang Xu, Yaqi Ren, Xiaolei Bai, Xinping Zhang

**Affiliations:** 1 School of Physical Science and Technology, Inner Mongolia University, Hohhot 010021, China; wangmeng@imu.edu.cn (M.W.); xuzhuangzhuang@mail.imu.edu.cn (Z.X.); renyaqi@mail.imu.edu.cn (Y.R.); baixiaolei@imu.edu.cn (X.B.); 2 School of Physics and Optoelectronic Engineering, Beijing University of Technology, Beijing 100124, China

**Keywords:** thin-film amplifier, distributed feedback, optical microcavity, fiber, polymer

## Abstract

We report a thin-film optical amplifier integrated on a fiber facet based on polymer-coated distributed feedback (DFB) microcavities, which are fabricated on a planar substrate and then transferred onto fiber tips by means of a flexible transfer technique. The amplified light directly couples into the fiber and is detected when coupled out at the other end after propagating along the fiber for about 20 cm. A prominently amplification factor of about 4.33 at 578.57 nm is achieved by sending supercontinuum pulses into the hundreds of micrometers’ DFB microcavities along the normal direction, which is also the axis direction of the fiber. The random distortions of grating lines generated during the transfer process result in a larger amplification spectral range and a less strict polarization dependence for injected light. Benefitting from the device size of hundreds of micrometers and the ease of integration, polymer amplifiers based on DFB microcavities demonstrate significant application potentials in optical communication systems and miniaturized optical devices.

## 1. Introduction

Due to their simple processing, high flexibility, low costs and ease of integration into compact devices, optical amplifiers based on polymer materials demonstrate great potential in integrated photonic and optoelectronic systems, covering both visible and near-infrared wavelengths to compensate for transmission losses [1,2,3,4,5,6]. Current studies on polymer optical amplifiers mainly focuses on waveguide amplifiers utilizing organic dye-doped and rare-earth ion-doped waveguides [7,8,9]. However, the spatial coupling of light from the small end face of the waveguide often leads to considerable coupling losses. Additionally, the amplification efficiency is highly dependent on the length of the waveguide, and several centimeters are typically required to achieve adequate gain performance. These limitations hinder the further integration, miniaturization, and practical application of the amplifier.

Microcavities, commonly used in polymer lasers [10,11,12,13], can also be used to create polymer optical amplifiers [14,15,16]. An efficient amplification and adjustment of amplification spectrum has been achieved based on distributed feedback (DFB) microcavities as reported in our previous works [14,16]. DFB resonators are the most widely used microcavities for its strong optical confinement, which enables a low-threshold and high-efficiency operation of the laser devices, even within a few hundred micrometers [17,18]. The vertical injection of DFB microcavities facilitates the integration of thin-film amplifiers with other optoelectronic devices through direct attachment to their surface, such as the end face of an optical fiber, the terminal face of a jumper plug, and can be combined with splitters, couplers, etc. The thin thickness of several hundred nanometers enables the optical amplifier to have a high integration level, with little to no impact on the original device’s performance and usage. The amplifier performance can also be easily modified, for instance, by employing different organic materials and DFB microcavity structures to alter the resonance wavelength, amplification linewidth and tunability [16]. The removal and replacement of such amplifiers are facile; for example, they can be wiped away by using a lens wiping paper dipped with acetone. Other types of gain mediums, such as perovskite [1,13], rare-earth-doped organic thin films [6,8], and colloidal quantum dots [19], may also be used to fabricate DFB microcavity amplifiers, thereby altering device performance and expanding application possibilities. The significantly amplified capability, the vertical injection method, the simplicity in altering performance and substitutability, and ultra-thin thickness in the transmission direction of DFB microcavities make them highly suitable for use as amplifiers in optical communication and integration with other optical devices.

In this work, thin-film DFB microcavity amplifiers integrated on a fiber facet were successfully fabricated using a flexible transfer technique and prominent amplification was achieved within hundreds of micrometers in size. The amplified light (AL) is directly coupled into the fiber and detected when it is coupled out at the other end after propagating along the fiber for a long distance. The random distortions of grating lines during the transfer process result in a broader amplification spectrum and disrupt the strict polarization dependence of the amplification of injected light. The miniaturization of the amplifier is greatly improved compared with waveguide amplifiers, with micrometer-scale device dimensions and nanometer-scale thickness of the DFB microcavities. The peeled-off DFB microcavities can be flexibly placed in any position to easily integrate with other optical devices. The light, when coupled into or outputting from a device, can be coupled into the DFB microcavities for amplification, which is very convenient in practical applications.

## 2. Design and Fabrication of the Organic Microcavity Laser Amplifier Integrated on Fiber Facet

**Figure 1:**
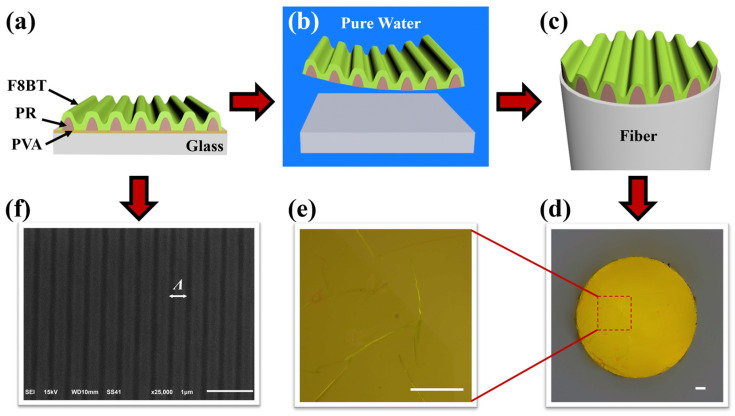
Fabrication procedures for the DFB microcavities integrated onto fiber facet. (**a**) DFB microcavities fabricated on glass substrate and separated by a layer of PVA film. (**b**) Liftoff of the DFB microcavities in pure water. (**c**) Pickup the DFB microcavities with a fiber. (**d**) Optical microscopic image of the DFB microcavities integrated on the fiber facet. (**e**) A high-magnification image of the area boxed in red in (**d**), and the scalebar is 50 μm. (**f**) A top-view SEM image of the DFB microcavities.

Figure 1a–c schematically illustrate the preparation processes of the DFB amplifier integrated onto a fiber facet. The DFB microcavities in Figure 1a, composed of polymer film and photoresist (PR, AR-P3170 from Allresist GmbH, Strausberg, Germany) gratings, were first produced on a glass substrate spin-coated with a layer of polyvinyl alcohol (PVA, with a concentration of 40 mg/mL in water) film. PR grating structures with a diameter of approximately 10 mm were created using interference lithography [19] on the substrate. The light-emitting polymer semiconductor poly(9,9-dioctylfluorenyl-2,7-diyl)-alt-co-(1,4-benzo-{2,1′,3}-thiadiazole) (F8BT, from American Dye Sources Inc., Quebec, Canada), dissolved in chloroform at a concentration of 23 mg/mL, was spin-coated onto the PR gratings with a rotational speed of 2500 rpm and a duration of 30 s. F8BT exhibits high electroluminescence (EL) and photoluminescence (PL) efficiencies and has been widely used in polymer lasers and amplifiers [2,14,16,17,20,21]. A top-view scanning electron microscopic (SEM, JSM-6510 from JEOL Ltd., Tokyo, Japan) image of the DFB microcavities fabricated above is shown in Figure 1f, revealing a grating period of approximately 368 nm. The resonant wavelength was calculated by using FDTD Solutions, which was approximately 578.6 nm as shown in Figure S1a, and the electric fields distribution of the resonant wavelength was shown in Figure S1b.

Before immersing the DFB microcavities in pure water, they can be cut to the desired size and shape using a knife. The water infiltrates from the cutting edge and dissolves the bottom layer of PVA, and the cut portion of the DFB microcavities detach from the glass substrate as shown in Figure 1b. Then, the hydrophobic DFB microcavities spread out on the water surface. A clean fiber was immersed in water to pick up the floating DFB microcavities from below, ensuring the fiber was in contact with the flat PR surface. Therefore, the fiber-integrated amplifier was constructed as shown in Figure 1c. Due to the small area of the fiber facet, the 10 mm diameter DFB microcavities can be cut into several small pieces with almost identical parameters and transferred onto multiple different fiber facets or other types of substrates. Finally, the transferred fiber was annealed at 60 °C to accelerate the evaporation of water and the dried DFB microcavities were firmly fixed onto the fiber facet. Figure 1d,e show the overall view and locally magnified optical microscopy images of the DFB microcavities after being transferred onto the fiber facet. The fiber we used is a coreless fiber with a cladding dimension of approximately 600 μm. Compared to traditional single-mode or multi-mode fibers, it has a larger cross-sectional area, making it easier to couple spatial light in our experiment. Moreover, during testing, the 600 μm coreless fiber is less susceptible to external vibrations and air currents due to its larger rigidity.

As shown in Figure 1e, the DFB microcavities exhibit random distortions and overlaps after transfer, which can lead to some changes in the performance of the amplifier compared to that on the planar substrate before transfer. But the majority of the transferred film remains smooth, with unchanged grating periods and uniform grating orientation. The variations in period of the DFB microcavities due to distortions and overlaps are limited, resulting in minimal shifts in resonant wavelengths, only a slight expansion in spectrum. And the lift-off procedure in water had no detrimental effect on the photoluminescence quantum yield of the F8BT film [20]. The preparation process is repeatable and reliable and leads to minimal discrepancies in the overall performance of the amplifiers. To reduce the impact of the transfer process on the properties of microcavities, a polyethylene terephthalate (PET) plate with a hole in its center can be employed as a frame to pick up the floating DFB microcavities from water, and then a fiber passes through the small hole to construct the fiber-integrated amplifier [17]. However, this method requires the DFB microcavities to be significantly larger than the supporting hole of the PET plate, which typically measures several millimeters in diameter. Compared to the diameter of fiber facet, a substantial portion of the structure is underutilized, thereby increasing the overall cost. Moreover, this design is unsuitable for transfer to other optoelectronic devices that cannot pass through the supporting hole.

## 3. Principle of the DFB Microcavity Amplifier

The DFB microcavities can function either as a DFB oscillator or as an amplifier, grounded in similar principles involving a second-order Bragg condition. The F8BT film serves as the gain medium and the periodic wrinkly structures modeled by the PR gratings act as the DFB microcavities, supporting stimulated emission amplification and waveguide mode resonance. In DFB microcavities, the wavelength *λ* of the waveguide mode resonated satisfied as follows:

2*n*_eff_*Λ* = *mλ*, (1)

where *n*_eff_ represents the effective refractive index, *Λ* denotes the grating period, and *m* is the order of the Bragg condition. The DFB microcavities in this work were also grounded in a second-order Bragg condition, where *m* equated to 2, and the laser was emitted perpendicularly to the surface. The resonance wavelength, *λ,* in the microcavities also satisfied the basic formula for grating diffraction:

*Λ* (*n*_1_ sin*i* ± *n*_2_ sin*r*) = ±k*λ.* (2)

As a DFB oscillator, the propagating and counterpropagating waveguide modes oscillate with an equivalent incident angle *i* of 90° in microcavities when *n*_1_ = *n*_eff_ and the laser output is directly to space with *n*_2_ = 1 at a diffractive angle of *r*, which is almost 0° for surface emission [22,23,24,25]. Consequently, the diffraction order, *k,* is equal to 1. The diffraction formula is transformed into *n*_eff_*Λ* = *λ*, which is exactly the second-order Bragg condition.

Acting as the DFB microcavity amplifier, the signal light injected should be coupled into the microcavities to form new waveguide modes that resonate and acutely interact with excited molecules through stimulated radiated emission. With the same resonance wavelength, *λ*, effective refractive index *n*_eff_ and period *Λ*, the available light injection methods are significantly constrained due to the limitations of the DFB microcavity structures. Along the opposite output route with an incident angle of 0°, the injected light was diffracted and coupled into the microcavities to form resonant waveguide modes. By slightly adjusting the incident angle around 0°, the main wavelength of the resonant waveguide modes can be adjusted within a highly restricted spectral range, as the diffractive angle remains constant for coupling in DFB microcavities. With a further increased incident angle, the waveguide resonance mode splits into two waveguide modes according to the basic formula of grating diffraction. The blue-shifted and red-shifted wavelengths do not satisfy the Bragg condition and significantly reduce the gain competition with oscillating light in the gain medium, rendering the injection unable to be amplified even with a higher pump fluence.

The injected light also can be coupled into the microcavities along the side edge perpendicular to the grating lines with an incident angle *i* of 90°. However, due to the hundred-nanometer thickness of the microcavities, side injection presents significant challenges and causes considerable losses. More importantly, side injection is not compatible with fibers for an end-facet-integrated amplifier. As a result, we adopt the normal incident method in our study, where the injection propagates along the axis of fiber and is amplified when passing through the DFB microcavities integrated on the end facet of fiber. The amplified light was coupled directly into the fiber and detected at the other end when coupled out.

## 4. The Performance of the DFB Microcavity Amplifier

**Figure 2:**
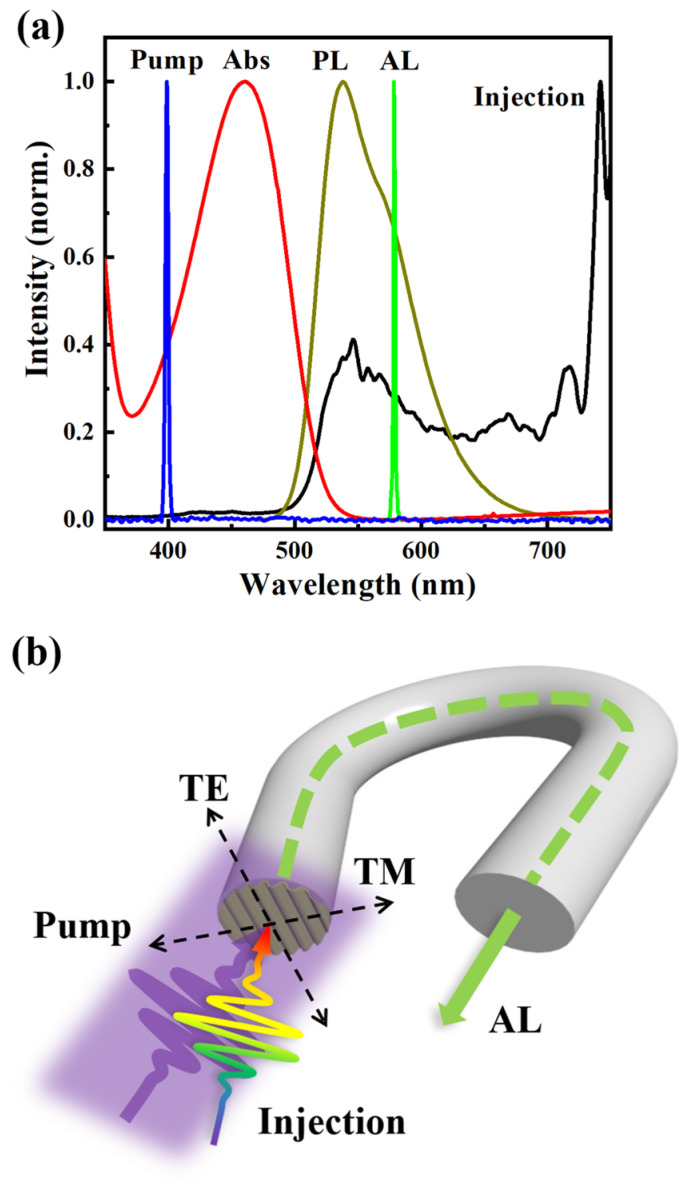
(**a**) The plots of the pump spectrum (blue), the injection white-light supercontinuum spectrum (black), the absorption spectrum of F8BT (red), the PL spectrum of F8BT (dark yellow), the amplified light spectrum (green) of the DFB microcavity, which are normalized individually and then combined. (**b**) The schematic illustration of the geometry of the DFB microcavity amplifier integrated on fiber facet.

The amplification performance of the DFB microcavity amplifier, integrated on a fiber facet, was investigated using a femtosecond pump–probe system. This system supplied both the pump and injection sources simultaneously, enabling the exploration of the amplification process dynamics in the femtosecond time scale. The femtosecond laser pulses were generated from a Ti/sapphire amplifier (Legend Elite from Coherent Inc., Santa Clara, CA, the United States of America) with a pulse length of approximately 150 fs at 800 nm. A significant portion of the 800 nm laser pulses was frequency doubled to 400 nm by a BBO crystal and used as the pump for the amplifier. The remaining portion was utilized to produce supercontinuum white light, serving as an injection, by sending to a 3 mm thick heavy water cell. The broad-band injected light is effective in obtaining the amplified spectrum range of the amplifier to replace a tunable light source and parts of the spectrum are shown in Figure 2a together with the pump and AL spectrum. The absorption and PL spectra of F8BT are also added in Figure 2a to show the relationship among all spectra involved in the amplification process. It should be noted that each spectrum in Figure 2a is individually normalized before being combined. The AL spectrum does not overlap with the absorption spectrum of F8BT, so the absorption loss of the material can be neglected when calculating the amplification factor. Although the injection light and the PL spectrum of F8BT have a large overlapping range, the full width at half maximum (FWHM) of the AL spectrum is small, which also proves the important role of the microcavity structure in the amplification process.

A schematic diagram for the measurements on amplification process is presented in Figure 2b. The injected light, with a diameter of approximately 200 μm, was coupled into the fiber along the fiber axis, while it was also sent to the DFB microcavities along the normal angle. The pump pulses were sent to the fiber facet at an angle of approximately 30° and were optimized to efficiently excite the entire DFB microcavities, with a larger diameter of approximately 2 mm compared to that of the fiber, ensuring the spatial overlap between the pump and injection laser beams. The amplified light was detected and characterized at the opposite end of the fiber, as illustrated by the green arrow passing through the fiber. The black arrow with dotted lines labeled “TE” and “TM” refer to polarization parallel and perpendicular to the grating lines of pump and injection pulses, respectively.

**Figure 3:**
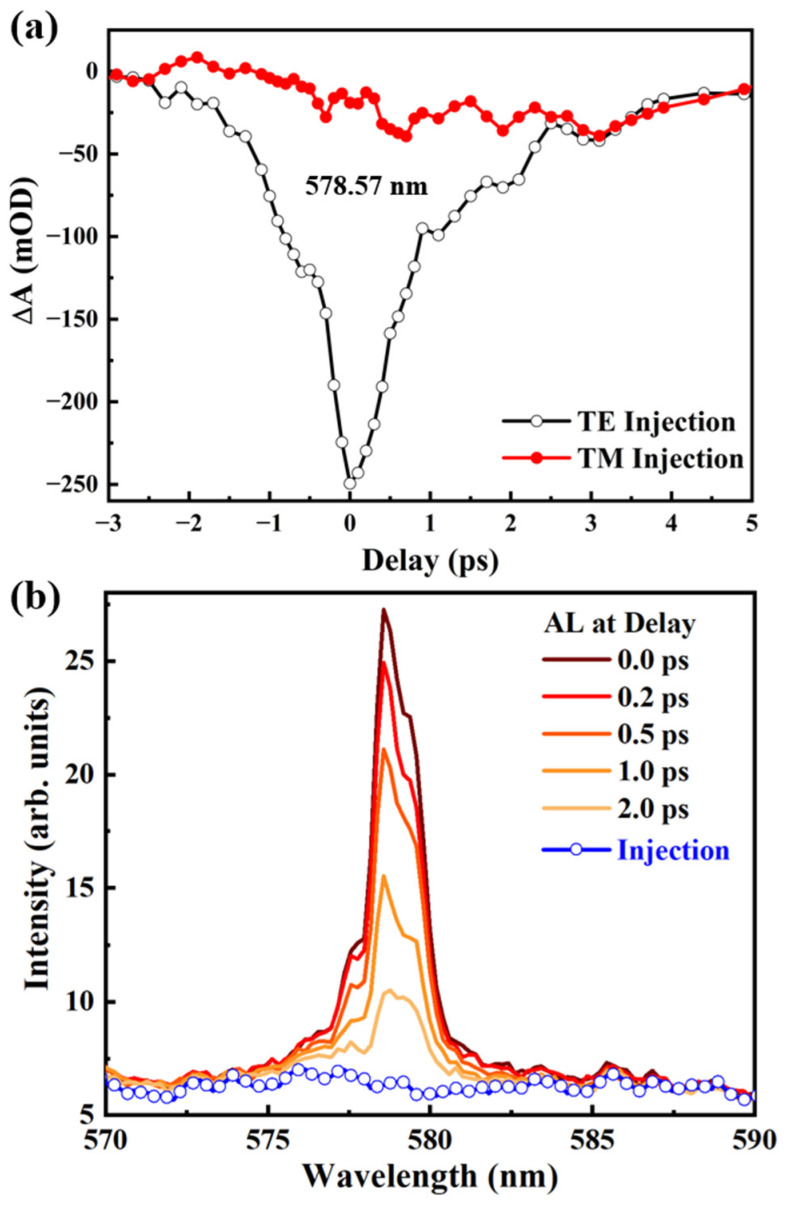
(**a**) Amplification dynamics of the injected light at 578.57 nm with TE (empty white circles) and TM (filled red circles) polarization as a function of delay time. (**b**) Injection (empty blue circles) and amplified light spectrum (red lines) at different time delay from 0 to 2.0 ps.

As the excitons decay rapidly after excitation, the time delay between the pump and injection significantly influences the amplification process. The transient amplification of the injected light was measured with a fixed pump fluence of about 20 μJ/cm^2^ at TE polarized for all measurements, unless otherwise specified. The results for 578.57 nm with different polarized injections are plotted in Figure 3a, showing a maximum amplification signal about −247 mOD for TE-polarized injection at a time delay of 0 ps. The varied transient absorption intensity implies a significant amplification for the injected light within a narrow time scale of 1–1.5 ps, which is mainly determined by the lifetime of the stimulated emission process [14,16]. The results also show a weak signal about −40 mOD for TM-polarized injection, which is inconsistent with our previous work. The difference in refractive indices for TE- and TM-polarized injections leads to different coupling wavelengths and coupling efficiencies. The TM-polarized injection cannot be amplified due to the mismatch of wavelength with the DFB oscillator, which was verified in our previous work [14]. Here, the DFB microcavities undergo random distortions during the transfer process onto fiber facet, and the tortile grating lines hinder the precise polarization and period. This leads not only to the weak amplification of TM injection but also a broadened amplified range in the spectrum compared to that on the planar substrate. As shown in Figure 3b, the real-time spectrum of the amplified light at different time delays was measured by a USB 4000 spectrometer from Ocean Optics, with the same detection head fixed behind the fiber and an integration time of 100 ms. The FWHM of the amplified spectrum is approximately 1.5 nm. As the time delay increases from 0 to 2 ps, a noticeable reduction in intensity can be observed, which is consistent with the transient absorption dynamics process described above. The maximum amplification factor is calculated as *I*_AL_/*I*_Injection_ at a time delay of 0 ps, and the corresponding values at 577.56 nm, 578.57 nm, and 579.38 nm are 1.81, 4.33, and 3.62, respectively. Furthermore, the peak of the AL at 578.57 nm decreases more rapidly than the peak at 579.38 nm, indicating a gain competition among different wavelengths for the injection.

**Figure 4:**
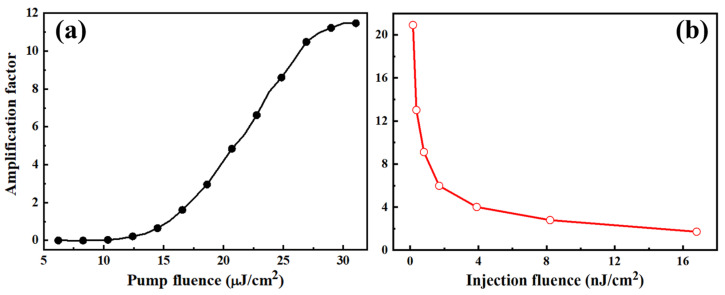
The relationship between the amplification factor at 578.57 nm and the pump (**a**) and probe (**b**) fluence, respectively.

The amplification behavior of the DFB microcavities is also influenced by the pump or injection fluence. As the amplification factor at 578.57 nm shows in Figure 4a, when the injection fluence remains constant, the amplification factor can be improved by increasing the pump fluence, which results in more excitons generated and interacting with the injected photons. This enhances the stimulated emission amplification process. However, when the pump fluence exceeds 30 μJ/cm^2^, the amplification factor remains constant due to the saturation of the interaction between the injected photons and the excitons. Taking into account the potential damage that higher pump fluence could cause to polymer molecules, a mild pump with a fluence value of 20 μJ/cm^2^ was used for all measurements. When the injection fluence is changed alone, the amplification factor varies, as shown in Figure 4b. Although the AL intensity improves with a higher injection fluence, the amplification factor decreases due to a slower increase in the AL intensity compared to the increase in the injection intensity. It should be noted that the fluence of the injection at 578.57 nm is too weak to be evaluated precisely, and the value here is roughly calculated by converting the spectrum intensity at 578.57 nm in the total supercontinuum spectrum [16].

## 5. Conclusions

Using a flexible transfer technique, the ultrathin DFB microcavities fabricated on planar substrates were successfully integrated onto a fiber facet. When light is coupled into the fiber with a normal incident angle, it can be amplified simultaneously by coupling into second-order DFB microcavities. The amplified light can transmit over a long range in fiber and be detected at the other end when coupled out. The random distortions of grating lines increase the FWHM of the amplification spectrum to about 1.5 nm and reduce the restriction of the amplifier on the injection polarization direction. An amplification factor of about 4.33 at 578.57 nm is achieved with a pump fluence of 20 μJ/cm^2^. The device size of hundreds of micrometers and the ease of integration make polymer DFB microcavity amplifiers potentially important for practical applications, such as in fiber communication, visible light wireless communication, photonic integrated circuits, chip-level monolithic photonic systems and flexible wearable devices.

## Data Availability

The data are available from the corresponding author upon reasonable request.

## Supplementary Materials

The following supporting information can be downloaded at: https://www.mdpi.com/article/10.3390/nano14151314/s1, Figure S1: The absorption spectrum and electric fields distribution of resonant wavelength of the DFB microcavities calculated by FDTD Solutions.
