# Supplementary material for: An Organic Microcavity Laser Amplifier Integrated on the End Facet of an Optical Fiber
Source: Nanomaterials (Basel). 2024 Aug 4;14(15):1314. doi: 10.3390/nano14151314 (PMC11313935; doi:10.3390/nano14151314)
Supplement: Supplementary file 1 [file nanomaterials-14-01314-s001.zip › nanomaterials-3120582-supplementary.pdf]

Organic microcavity laser amplifier integrated on the end facet of an optical fiber

Meng Wang [1], Zhuangzhuang Xu [1], Yaqi Ren [1], Xiaolei Bai [1] and Xinping Zhang [2,*]

[1] School of Physical Science and Technology, Inner Mongolia University, Hohhot, Inner Mongolia 010021, China 1; wangmeng@imu.edu.cn (M.W.); xuzhuangzhuang@mail.imu.edu.cn (Z.X.); renyaqi@mail.imu.edu.cn (Y.R.); baixiaolei@imu.edu.cn (X.B.)
[2] School of Physics and Optoelectronic Engineering, Beijing University of Technology, Beijing 100124, China 2;
* Correspondence: zhangxinping@bjut.edu.cn (X.Z.)

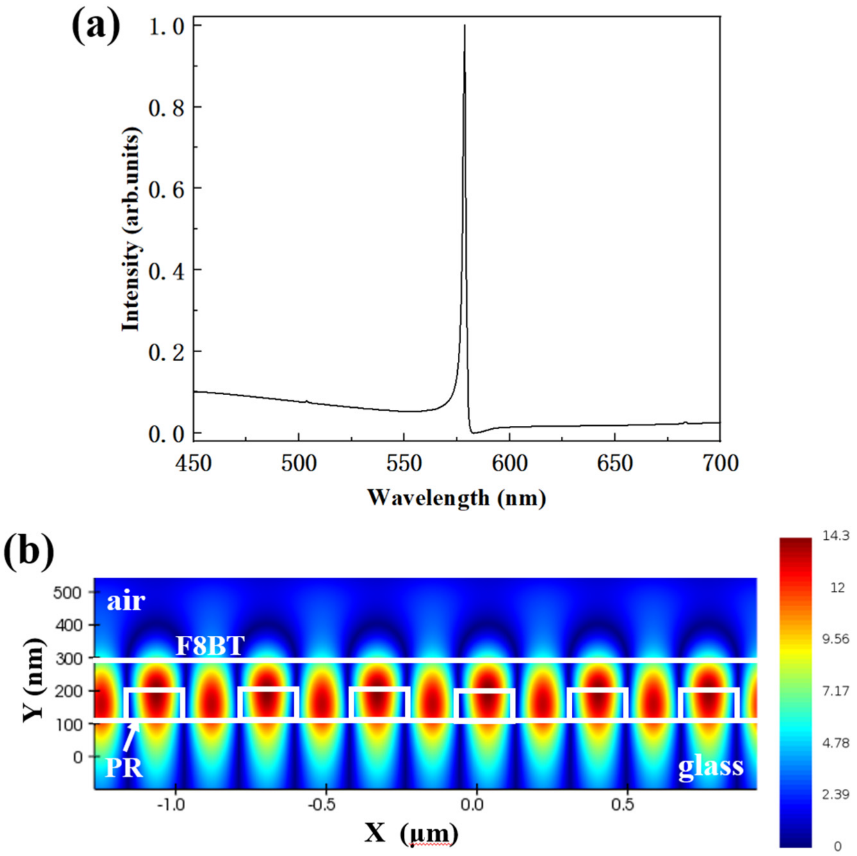

Figure S1 (a) The normalized absorption spectrum and (b) the electric fields distribution of resonant wavelength of the DFB microcavities calculated by FDTD Solutions.
